# Depression, Stress and Anxiety among Women and Men Affected by Recurrent Pregnancy Loss (RPL): A Systematic Review and Meta-Analysis

**DOI:** 10.3390/life13061268

**Published:** 2023-05-27

**Authors:** Annalisa Inversetti, Giampaolo Perna, Gloria Lalli, Giuseppe Grande, Nicoletta Di Simone

**Affiliations:** 1 Humanitas University Department of Biomedical Sciences, Humanitas University, Via Rita Levi Montalcini 4, 20072 Pieve Emanuele, Milan, Italygloria.lalli@st.hunimed.eu (G.L.); nicoletta.disimone@hunimed.eu (N.D.S.); 2 IRCCS Humanitas Research Hospital, Via Manzoni 56, 20089 Rozzano, Milan, Italy; 3 Unit of Andrology and Reproductive Medicine, Department of Medicine, University of Padova, 35128 Padua, Padua, Italy; grandegius@gmail.com

**Keywords:** recurrent pregnancy loss, depression, stress, anxiety

## Abstract

The aim of the present study is to perform a systematic review and meta-analysis on depression, stress and anxiety in women who experienced recurrent pregnancy loss (RPL) compared to controls and to men who experienced RPL. The pooled results showed a higher level of moderate/severe depression among women who experienced RPL compared to controls (5359 women, random effects model, odds ratio (OR) 3.77, 95% Confidence Interval (CI) 2.71–5.23, *p* < 0.00001, I^2^ 0%). Anxiety and stress levels were also higher among women experiencing RPL compared to controls. The pooled results showed a higher level of moderate/severe depression in women who experienced RPL compared to men who underwent the same experience (113/577 (19.5%) women versus 33/446 (7%) men versus random effects model, OR 4.63; 95% CI 2.95–7.25, *p* < 0.00001 I^2^ 0%). Similarly, higher levels of stress and anxiety in women experiencing RPL compared to men experiencing RPL were described. Women who experienced RPL showed higher rates of moderate–severe depression, stress and anxiety compared to both controls and men who experienced RPL. Healthcare professionals should implement screening for anxiety and depression and social support for both partners and support them in dealing with RPL according to sex-specific responses to this stressful event.

## 1. Introduction

Recurrent pregnancy loss (RPL), defined as the spontaneous loss of two or more pregnancies (according to the American Society for Reproductive Medicine), presents several still incompletely defined aspects.

Several studies report that women who have experienced a single pregnancy loss had increased rates of stress, anxiety and depression [1]. For some of them, symptoms persisted for longer, even one year after the event [2]. Some authors observed that negative psychological effects and feelings of grief related to abortion can be more intense when the event is recurrent [3,4]. Recent studies showed an increased prevalence of depressive symptoms and psychiatric diagnoses among women suffering from RPL [5], and this was particularly evident when compared with other women trying to conceive [6].

Regarding men’s mental health, it has previously been shown that pregnancy loss also affects them emotionally [7,8]. However, less attention is given to this specific issue compared to women and men’s reactions to their partner’s experience have been not investigated for many years. In a controlled follow-up study by Beutel and colleagues [9], 56 couples were studied immediately after one miscarriage, and then 6 and 12 months later. The participants completed standardized questionnaires for depression, physical complaints, anxiety and grief. The results showed that men who experienced RPL suffered less intensely and for less time compared to their partners. The way in which they experience grief was similar to that of women, except that men felt less need to share their feelings with experts or with their partners. In contrast to women, they did exhibit an increased depressive reaction (compared to age- and sex-matched community control groups).

In a recent qualitative study, men described feeling external pressure to maintain a positive attitude and support their partners despite their own feelings of loss after a pregnancy loss [10]. In general, concerning mental illnesses, it has been argued that the burden of the diseases is generally underestimated [11]. Vigo et al. hypothesized five causes for this underestimation that range from the overlap between psychiatric and neurological disorders to the exclusion of personality disorders from disease burden calculations. The psychological effect of RPL on the male partner has begun to be further explored only in recent years, as well as the correlation between depression and emotional stress within the couples who have experienced RPL. However, the different psychological impact on women compared to men after RPL has not yet been subject to a systematic review of the literature available.

The aim of the present study is to perform a systematic review and metanalysis on depression, stress and anxiety among women who experienced RPL compared to both controls and men who experienced RPL.

## 2. Materials and Methods

This study was carried out according to the PRISMA guidelines for systematic reviews.

### 2.1. Search Strategy

A systematic literature search was performed in PubMed/MEDLINE, SCOPUS, Embase and Web of Science to identify studies published from 1980 to February 2022. The electronic search strategy included the following keywords: depression OR depressive symptoms OR major depression OR stress OR anxiety OR anxious disorder OR stress OR psychological impact OR psychological effect AND recurrent pregnancy loss OR habitual abortion OR recurrent miscarriage. Reference lists and topic-related reviews were manually searched to identify further relevant papers.

### 2.2. Selection of Studies

The studies were selected by screening titles and abstracts, and full-text copies of those eligible were further assessed independently by two investigators (A.I. and N.D.S.) according to the inclusion and exclusion criteria described below. In case of overlapping studies, only the largest and most complete dataset was included.

### 2.3. Inclusion and Exclusion Criteria

We retrieved all randomized controlled trials (RCT), clinical controlled trials, retrospective and prospective cohort studies and cross-sectional and case–control studies on the topic described above. We applied a language restriction to English studies. A “PICO” (Patient–Intervention–Comparison–Outcome) of interventional studies was used to define the specific questions to be assessed, in particular:

- Participants/population: women;
- First exposure: RPL;
- First Comparator: women not affected by RPL but who are trying to conceive (defined as “controls”);
- Second comparator/control: men who experienced RPL;
- Primary outcome(s): moderate/severe depression;
- Secondary outcomes: stress and anxiety.

We excluded duplicates, reviews, case reports, incomplete reports, book chapters, conference abstracts, letters to the editor and comments. We included only studies in which the experimental group had at least two or more consecutive pregnancy losses and depression and anxiety clearly defined by validated tools, and studies in which a group for comparison (either men who experienced RPL or controls) was included.

### 2.4. Quality Assessment of Studies

The results of our risk assessment are summarized in Tables 3 and 4. We used the Newcastle–Ottawa Assessment scale [12] customized for cross-sectional studies. This scale has a scoring system using asterisks based on three domains, including the selection of study groups, the comparability of groups and ascertainment of exposure. A maximum of five asterisks could be given to the selection domain (if the sample is truly representative of the average in the target population), two asterisks to the comparability domain (if the subjects in different study groups are comparable based on the study design or analysis) and three asterisks to the exposure domain (if there is a reliable ascertainment of the outcome and a clearly described, appropriate statistical test). A greater number of asterisks indicates a greater quality.

We also graded the quality of evidence using the Grading of Recommendations Assessment, Development and Evaluation (GRADE) approach [13].

### 2.5. Presentation of Data

Extracted data from all the included articles are displayed in Table 1 and Table 2.

### 2.6. Statistical Analysis

The meta-analysis was performed to estimate the pooled odds ratio (OR) with a 95% confidence interval (CI) using the random effect model. The statistical heterogeneity among studies was tested with the *I^2^* test. We combined each outcome and calculated a summary effect size using the statistical software Review Manager 5.4 (Computer Program) according to guidance from the Cochrane Handbook.

## 3. Results

### 3.1. Search Result

We performed search strategies which led to a set of results that were selected for two meta-analyses: the first on the comparison of depression between women affected by RPL and controls (women who did not experience RPL, but are trying to conceive) and the second comparing women affected by RPL to men who experienced RPL in the couple.

**Figure 1:**
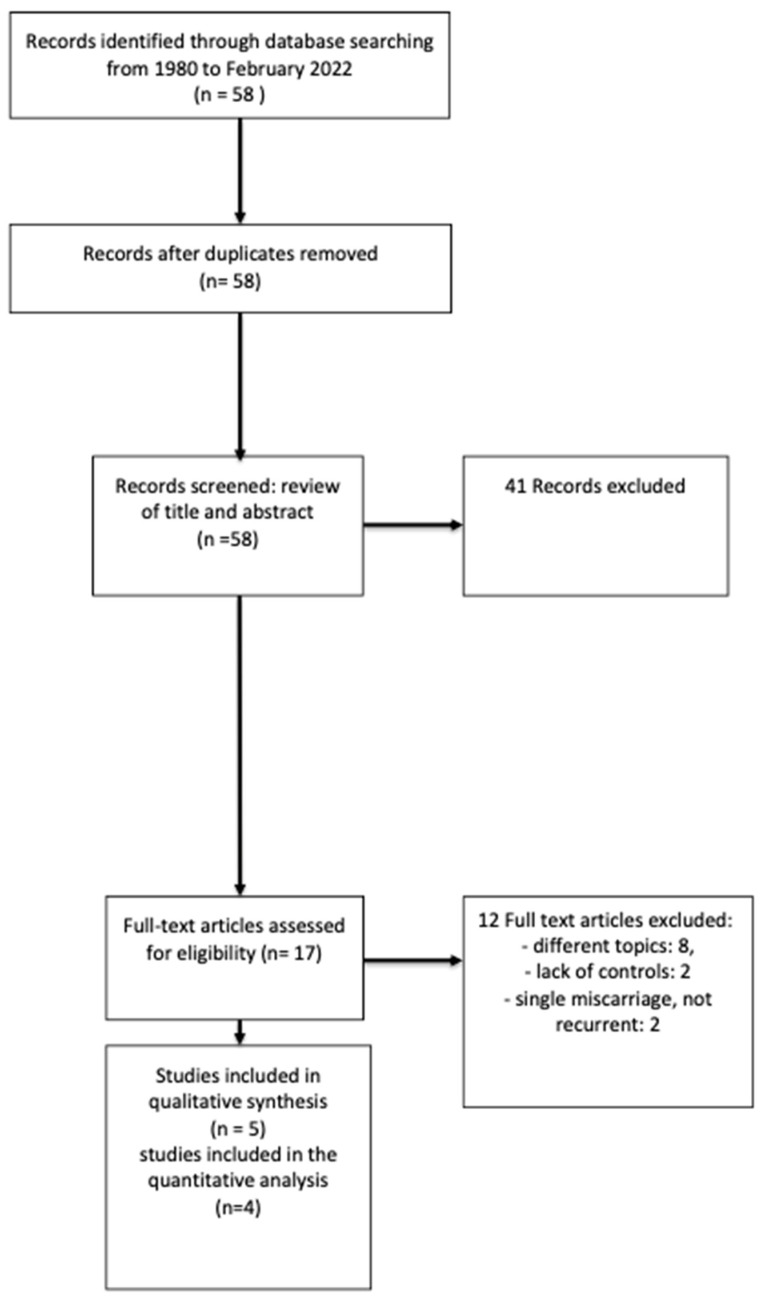
PRISMA flow chart on depression in women affected by RPL and controls.

**Figure 2:**
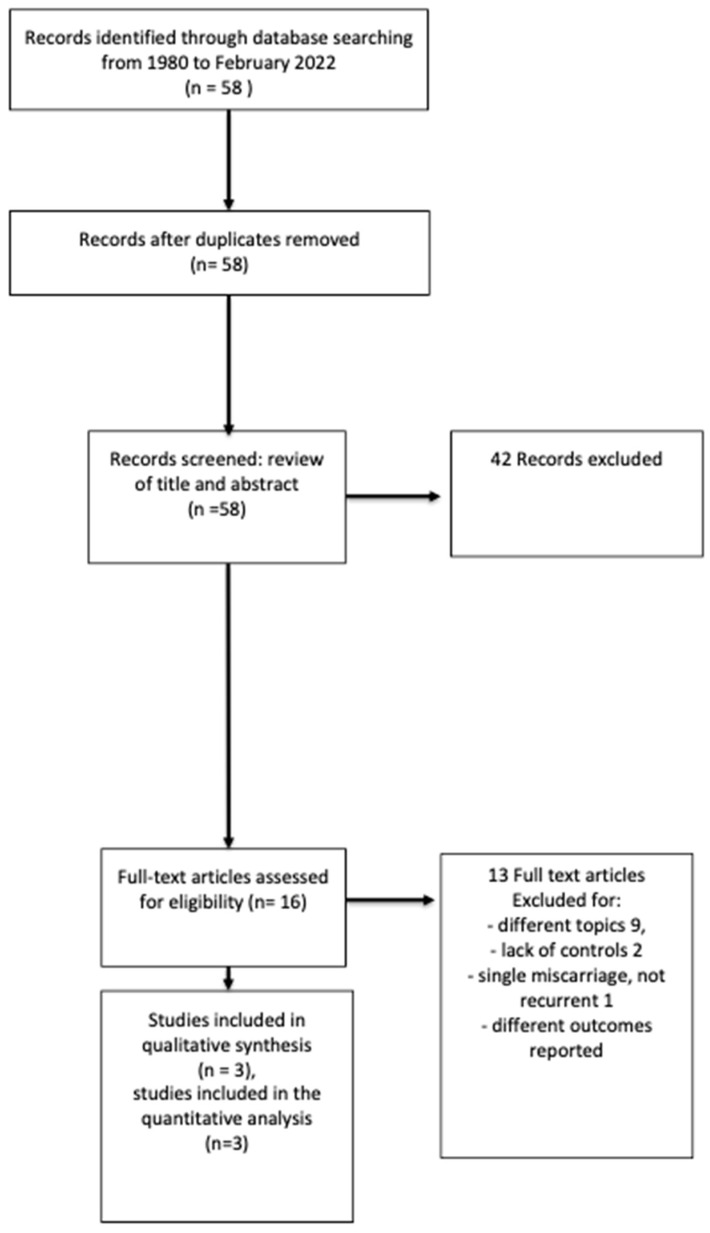
PRISMA flow chart on depression in women and men affected by RPL.

(1) 58 studies were identified by the search strategy and screened for titles and abstracts. A total of 41 were excluded (details in Figure 1). Full-text articles of seventeen studies were assessed for eligibility, of which five were included in the qualitative and four in the quantitative analyses. We excluded one study [17] from the meta-analysis due to the control group: women with one miscarriage rather than women without a history of miscarriage.
(2) 58 studies were identified by the search strategy and screened for titles and abstracts. A total of 42 were excluded (details in Figure 2). Full-text articles of three studies were assessed for eligibility, of which three were included in the qualitative and quantitative analyses.

Regarding the secondary outcomes (stress and anxiety), we decided to perform only a systematic review and narrative description of the results, without pooling the results in a meta-analysis because only a small number (<3) of studies reported on these outcomes comparing women experiencing RPL to controls. Thus, we judged it would not be informative to perform a quantitative analysis.

### 3.2. Quality Assessment

The risk of bias and quality assessment results are summarized in Table 3. Amongst the ten applicable stars assessing the three main categories of selection, comparability and outcomes, the eligible studies received between eight and nine stars. No study received the maximum score (10 stars) because the outcomes were based on self-reports and not on objective measurements.

Furthermore, in terms of the selection of the study population, we judged all representatives, except for one [19] that chose a sample with a higher than average educational level, but the authors discussed this topic in the limitations of the study. In terms of response rate, it was high in almost every study included, ranging from 66% to 94%. Regarding the ascertainment of the exposure, two studies [15,18] used depression and anxiety scores (SDS and SAS) that were not validated specifically in the RPL population. The validated ones were Cohen’s Perceived Stress Scale (PSS) and the Major Depression Index (MDI).

Comparability and statistical tests were of high quality in all the included studies.

A summary of the quality of evidence according to the GRADE system is reported in Table 4. Owing to the design of the included studies, the quality of evidence was low to very low.

### 3.3. Quantitative Analysis—Meta-Analysis on Moderate/Severe Depression in Women Experiencing RPL versus Controls and Women Experiencing RPL versus RPL Men

**Figure 3:**
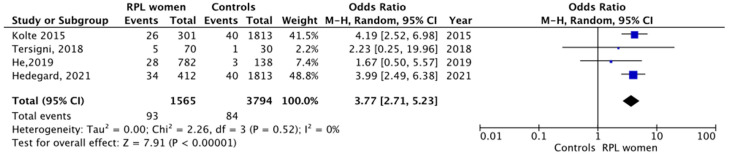
Forest plot on depression level in women experiencing RPL versus controls [6,14,15,16].

From a total of 58 initially identified, 5 studies were included in the qualitative comparison between women who experienced RPL compared to controls. However, only four studies met the criteria for the quantitative comparison. Pooled results from four studies (three cross-sectional and one case–control) showed a higher level of moderate/severe depression in women who experienced RPL compared to controls (93/1565 (5.9%) women experiencing RPL versus 84/3794 (2.2%) controls, random effects model, OR 3.77, 95% CI 2.71–5.23, *p* < 0.00001, I^2^ 0%, Figure 3).

**Figure 4:**
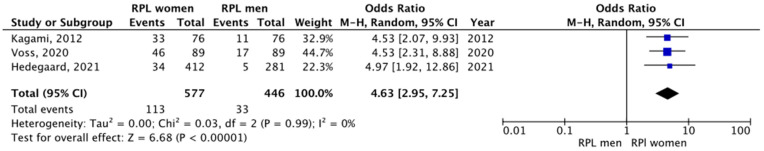
Forest plot on depression level in women experiencing RPL versus men [16,18,19].

Three studies were included in the comparison between women and men who experienced RPL. Pooled results from three cross-sectional studies showed a higher level of moderate/severe depression in women who experienced RPL compared to men who underwent the same experience (113/577 (19.5%) women versus men 33/446 (7%) men, random effects model, OR 4.63; 95% CI 2.95–7.25, *p* < 0.00001 I^2^ 0%, Figure 4).

### 3.4. Qualitative Analysis—Stress in Women Experiencing RPL Compared to Controls and Anxiety in Women Experiencing RPL Compared to Controls

Stress and anxiety levels in women experiencing RPL compared to controls were reported in two and three studies, respectively. Due to the low number of studies, we opted for a qualitative analysis and decided not to pool the results in a meta-analysis.

Higher stress levels in women experiencing RPL compared to controls were reported by Kolte et al. (42% among RPL versus 23.1%) [6] and Hedegaard et al. (28.6% versus 23.1%) [16]. They both used Cohen’s Perceived Stress Scale (PSS).

Regarding anxiety levels, in all the three studies on this topic, higher anxiety levels in women experiencing RPL compared to controls were reported. Tersigni et al. [14] evaluated the outcome through the STAI Y test, while both He et al. [15] and Wang et al. [17] used the SAS test (Self-Rating Anxiety Scale). In particular, Tersigni et al. [14] reported 4% of anxious women among those who experienced RPL compared to 1.4% in controls. He et al. [15] and Wang et al. [17] reported the same findings: 2% in RPL versus 1.4% in controls and 9.3% versus 5.4%, respectively.

### 3.5. Qualitative Analysis—Stress in Women Experiencing RPL Compared to Controls and Anxiety in Women Experiencing RPL Compared to RPL Men

Similarly, a higher level of stress in women experiencing RPL compared to men was shown by Hedegaard et al. [16] using the PSS (28.6% in women experiencing RPL versus 10.6% in men)*,* the only study reporting on this outcome in men. Women experiencing RPL were again more anxious if compared to men in two studies reporting on this outcome, both using the STAI test. Kagami et al. [18] and Voss et al. [19] described 25% of anxious women experiencing RPL versus only 4% of men, and 47.7% in women experiencing RPL compared to 19.1% in men, respectively.

## 4. Discussion

Women who experienced RPL showed higher rates of moderate–severe depression, stress and anxiety compared both to women who have not experienced RPL and to men who have experienced RPL. Our findings are in line with previous studies. Recent studies have already demonstrated that the psychological approach to pregnancy loss is gender specific [2,9,20]. Nonetheless, these observations were derived mainly from couples with a single miscarriage, and less is known about the impact of the event when is repeated. Furthermore, data regarding the perception of RPL and couples’ relationships are very recent and the topic has gained more attention only in recent years. In terms of the intensity of the distress caused by the event, it seems that women usually experience this feeling more strongly and at a deeper level than men [2]. Beutel et al. (1996) [9] reported that the levels of depression among women with a recent pregnancy loss were significantly increased compared to those of the matched women, whereas their partners’ levels of depression were not significantly higher compared to the controls. We can hypothesize that this difference between men and women might be due to using the same tool to assess depression in different sexes. In this context, we suggest there is a need for more interest in research on gender-tailored approaches to RPL. From a pathogenic point of view, Tersigni et al. [14] highlighted that depression and inflammation are linked and feed off each other. We can further hypothesize a tri-directional loop, in which RPL increases depression, which facilitates inflammatory responses, which then in turn promotes depression. Recent evidence [21] has corroborated the supposed associated effect of the innate and adaptive immunity in determining depression and inflammation. It is noteworthy that elevated inflammatory signaling dysregulates neurotransmitter release and causes changes in neuronal activity in specific brain regions involved in the pathogenesis of depression. In particular, it has been proven that an inflammatory state may be associated with depressive symptoms [22]. Conversely, depression is accompanied by an activation of the inflammatory response system. In the context of RPL, the condition of “leaky gut” could explain the increase in the inflammatory levels in the endometrium; an abnormal intestinal permeability allows the passage of antigens through the intestinal barrier, which might elicit innate immunity in the endometrial tissue [23]. Hence, when there is an increased intestinal permeability in RPL subjects, there might be a higher risk of endometrial inflammation and reproductive disorders. The onset of a link between the gut and the endometrium may be an interesting hypothesis that could suggest a possible new approach for a specific group of patients with idiopathic RPL [14,24,25]. Future research should evaluate the eventual association between leaky gut and depression in RPL women. Recent evidence has highlighted that depressive symptoms, if left untreated, can cause adverse outcomes in future pregnancies. In detail, Vlenterie et al. [26] suggested that depressive symptoms or a clinical diagnosis of depression during pregnancy are associated with preterm births and low Apgar scores, even without exposure to antidepressants. Furthermore, due to the fact that depression is associated with a decreased quality of life, postpartum depression and adverse pregnancy outcomes, pharmacological treatment might be recommended. Consequently, in recent years, antidepressant use among pregnant women has increased substantially, with the prevalence estimated between 1 and 8%. From the results of the individual participant data meta-analysis by Vlentierie et al., the authors concluded that a clinical diagnosis of depression during pregnancy should not be left untreated. Although other treatments may be preferred, pharmacologic treatment might be an option for women suffering from clinically diagnosed moderate to severe depression. Use of SSRIs, especially fluoxetine and sertraline, however, was also associated with increased risks of preterm birth and low Apgar scores. The results of this individual participant data meta-analysis may help healthcare professionals and pregnant women in making evidence-based decisions on whether the beneficial effects of pharmacologic treatment of maternal depression outweigh the possible risks for the fetus. Healthcare professionals should be aware of the risks of the underlying disorder itself and provide pregnant women with appropriate pharmacologic treatment when necessary. Concerning the limitations of the study, we first mention the heterogeneity of the tests used for assessing depression, stress and anxiety; not all were validated in the context of RPL. Second, we highlight the different definitions of RPL used in the studies (two previous pregnancy losses versus three previous pregnancy losses). Third, we also highlight the low quality of the studies (all observational, mainly cross-sectional and case–control studies). However, we should mention that some research questions cannot be addressed with quantitative methods. Focusing on the nature of the topics of our research, it should be underlined that the GRADE system, based on a rigorous evaluation mainly on the study design (RCTs are rated as high quality), has not reflected the value of this systematic review and meta-analysis from a qualitative point of view.

Fourth, the outcomes were all self-reported (not objective measurements) without a strong diagnostic validity. In particular, two of the included studies [6,16] pooled in the meta-analysis on depression in women experiencing RPL versus controls and versus men experiencing RPL had the same control group: women who were part of the Soon Parents Study (www.SnartForaeldre.dk) performed at the Department of Clinical Epidemiology, Aarhus University Hospital. This study included males and females who were actively trying to conceive, in a heterosexual relationship, not using contraception and not presently receiving fertility treatment. The included women were between 18 and 45 years. The Soon Parents Study was initiated in August 2011, and participants were recruited by press coverage and advertisements on selected websites popular among couples trying to conceive. However, the two studies considered different cases (women experiencing RPL); Kolte et al. recruited cases from 2010 to 2013 and Hedegaard et al. from 2015 and 2018. Thus, on this basis, we considered them as two different entities in the meta-analysis. To the best of our knowledge, this is the first meta-analysis on the psychological impact of RPL on men and comparing the effect between men and women. Second, we noticed a high response rate to the questionnaires administered. In all the included studies, statistical analyses were clearly explained. Our results are in line with previous studies and no less relevant.

## 5. Conclusions

This literature review and meta-analysis evaluated the psychological impact of RPL on women compared to men but also to women who have not experienced RPL. The findings highlighted that women affected by RPL showed higher rates of moderate–severe depression, stress and anxiety both compared to controls and to men who experienced RPL.

Since a gynecologist is in general the first healthcare provider to meet a couple who are affected by RPL, they should be aware of the psychological effects on both men and women, with the double aim of offering a more comprehensive support to the couple and refer both of them to a mental health specialist.

On the other side, the mental health specialist should tailor RPL counselling according to the gender of the patient, considering different psychological adjustments.

Given the higher presence of depression, anxiety and stress among women with RPL, a key aspect to clarify is the nature of these mental states. Are they physiological emotional reactions to repeated loss experiences or do they represent abnormal psychopathological phenomena? How can we distinguish them? What are the consequences of these states on the long-term mental health of parents and families? What might their effects be on the possibility of a favorable pregnancy? Are there risk factors for the development of these emotional states? Can we develop specific psychological protocols to help overcome these mental states? Are there more effective and appropriate drugs in these situations? These are some of the open questions that deserve attention from the scientific community given the need to reduce global psychological grief in couples who are experiencing a very stressful situation.

## Figures and Tables

**Table 1:** Main characteristics of the reviewed studies in the comparison between women experiencing RPL and controls.

Author, Year	Area	Design	Depression (Scale)	Stress (Scale)	Anxiety (Scale)	Cut Off for Depression	Cut Off for Stress	CUT Off for Anxiety	Administration of the Questionnaire	Controls	Definition of RPL	Depressed in RPL (n)	Total RPL (n)	Depressed in Controls (n)	Controls (n)	Stressed/Anxious in RPL (n)	Total RPL	Stressed/Anxious in Controls (n)	Controls (n)	Adjustments
Kolte, 2015 [6]	Denmark	Cross-sectional	Major Depression Index (MDI)	Cohen’s perceived Stress Scale (PSS)	/	Moderate * Severe **	Moderate/severe ≥19	/	Online questionnaire	Non pregnant women who are trying to conceive	Written questionnaire at first visit	26	301	40	1813	124 (Stress)	301	420 (Stress)	1813	Age, education, household income, number of children, prior pregnancies
Tersigni, 2018 [14]	Italy	Case-control	Zung Self-rating Depression Scale (Z-SDS)	/	STAI Y test	Moderate (60–69) Severe (≥70)	/	Moderate (50-60) Severe (>60)	Written questionnaire at first visit	Healthy women with two or more previous uncomplicated pregnancies	Written questionnaire at first visit	5	70	1	30	3 (Anxiety)	70	1 (Anxiety)	30	Age, BMI, IVF
He, 2019 [15]	China, Shanghai	Cross-sectional	Self-rating Depression Scale (SDS)	/	Self-rating Anxiety Scale (SAS)	Moderate (60–69) Severe (≥70)	/	Moderate (60–69) Severe (≥70)	Written questionnaire at first visit	Women with no previous pregnancy loss and not presently receiving any fertility treatment.	Two or more miscarriages before 24 weeks	28	782	3	138	16 (Anxiety)	782	2 (Anxiety)	138	Duration of marriage, household income, history of induced abortion and history of previous live birth
Hedegaard, 2021 [16]	Denmark	Cross-sectional	Major Depression Index (MDI)	PSS	/	Moderate * Severe **	Moderate/severe ≥19	/	Online questionnaire	Non pregnant women who are trying to conceive	Three or more consecutive miscarriages before 22 weeks	34	412	40	1813	110 (Stress)	384	420 (Stress)	1813	Age, number of losses and primary versus secondary recurrent pregnancy loss
Wang, 2021 [17]	China, Gansu region	Nested case–control	Self-rating Depression Scale (SDS)	/	Self-rating Anxiety Scale (SAS	Moderate (63–72) Severe (≥72)	/	Moderate (60–69) Severe (≥70)	Self-rating questionnaires + in-person structured interview	One previous miscarriage	Two or more miscarriages before 24 weeks	208	1132	194	1426	106 (Anxiety)	1132	77 (Anxiety)	1426	Age, ethnicity, education, family monthly income, active smoking, previous liveborn, and embryonic chromosome abnormalities

Note: * Moderate: two core symptoms and four or more additional symptoms, or three core symptoms and four additional symptoms. ** Severe: three core symptoms and five or more additional symptoms.

**Table 2:** Main characteristics of the reviewed studies considered in the comparison between women and men who experienced RPL.

Author, Year	Area of Study	Type of Study	Scale of Depression	Scale of Stress	Scale of Anxiety	Cut Off for Moderate/Severe Depression	Cut Off for High Stress	Cut Off for Anxiety	Administration of the Questionnaire	Comparison Group	Definition of RPL	Number of Depressed Men among RPL	Total RPLmen	Number of Depressed among RPL Women	Total RPL Women	Number of Anxious Men among RPL	Total Men rplRPL	Number of Anxious among RPL Women	Total RPL Women	Adjustment for Confounders
Kagami, 2012 [18]	Japan	Cross sectional	Beck depression inventory 2 ed. (BDI-II)	/	State Trait Anxiety Inventory (STAI)	Moderate (score: 20–28) Severe (score ≥29)	/	Score > 55	Written questionnaire at first visit (some completed at clinic, some at home)	Women	Two or more miscarriages before 22 weeks	11	76	33	76	3	76	19	76	Age, length of marriage, income, education, n of PL, previous live birth
Voss, 2020 [19]	Germany	Cross sectional	ScreenIVF from BDI	/	ScreenIVF from STAI	Score >4	/	Score > 24	Written questionnaire at first visit	Women	Two or more miscarriages before 22 weeks	17	89	46	89	17	89	42	88	Gender, number of PL, social support
Hedegaard, 2021 [16]	Denmark	Cross sectional	Major Depression Index (MDI)	Cohen’s perceived Stress Scale (PSS)	/	Moderate * Severe **	Score of ≥19 at PSS scale	/	Online questionnaire	Women	Three or more consecutive miscarriages before 22 weeks	5	281	34	412	30	281	110	384	Age, number of losses and primary versus secondary RPL

Note: * Moderate: two core symptoms and four or more additional symptoms, or three core symptoms and four additional symptoms. ** Severe: three core symptoms and five or more additional symptoms.

**Table 3:** Risk of bias and quality assessment using Newcastle–Ottawa Assessment scale.

Author, Year	Selection				Comparability	Outcome		Total Score
	Representativeness of the Sample	Sample Size	Non-Respondents	Ascertainment of the Exposure		Assessment of the Outcome	Statistical Test	
Kagami, 2012 [18]	*	*	* response rate 66%	* validated tools	** adjustment for many factors	* self-report (some at the clinic, some at home)	*	8/10
Kolte, 2015 [6]	*	*	* response rate 69%	** validated tools	**	* self-report online	*	9/10
Tersigni, 2018 [14]	*	*	not reported	** validated tools	*	**	*	8/10
He, 2019 [15]	*	*	* response rate 94.1%	* not validated on RPL or fertility population	**	* self-report at the clinic	*	8/10
Voss, 2020 [19]	0 Above average educational background	*	* response rate 76.4%	** validated tools on fertility, not all in RPL	**	* self-report at first visit	*	8/10
Hedegaard, 2021 [16]	*	*	* response rate 76% for both questionnaires	** validated tools	**	* self-report online	*	9/10

Note: * A maximum of five asterisks could be given to the selection domain (if the sample is truly representative of the average in the target population), two asterisks to the comparability domain (if the subjects in different study groups are comparable based on the study design or analysis) and three asterisks to the exposure domain (if there is a reliable ascertainment of the outcome and a clearly described, appropriate statistical test).

**Table 4:** Moderate/severe depression estimates according to RPL and sex. Summary of results and quality of evidence.

Comparator	No. of Studies	Study Design	Effect Estimate [95%CI]	Quality of Evidence (GRADE)
Moderate/severe depression in women experiencing RPL versus RPL men	3	Cross-sectional	OR 4.63 [2.95–7.25]	Low
Moderate/severe depression in women experiencing RPL versus non RPL women	4	Three Cross-sectional One Case-control	OR 3.77 [2.71–5.23]	Low

## Data Availability

Upon request.
